# The 25-year experience of over-the-top ACL reconstruction plus extra-articular lateral tenodesis with hamstring tendon grafts: the story so far

**DOI:** 10.1186/s40634-023-00599-8

**Published:** 2023-04-01

**Authors:** Stefano Zaffagnini, Gian Andrea Lucidi, Luca Macchiarola, Piero Agostinone, Maria Pia Neri, Maurilio Marcacci, Alberto Grassi

**Affiliations:** 1 Clinica Ortopedica E Traumatologica II, IRCCS Istituto Ortopedico Rizzoli, Via Giulio Cesare Pupilli, 1, Bologna, Italy; 2 Ortopedia E Traumatologia, IRCCS Casa Sollievo Della Sofferenza, San Giovanni Rotondo, Italy; 3 Department of Biomedical Sciences, Humanitas University, IRCCS Humanitas Clinical and Research Center, Milan, Italy

**Keywords:** Anterior Cruciate Ligament, Lateral Pasty, Over-the-Top, ACL, Pivot shift

## Abstract

This article presents with an evidence based approach, the kinematical rationale, biological evidence and the long term results of the “Over-The-Top” anterior cruciate ligament reconstruction with lateral plasty technique. This surgery was developed more than 25 years ago at the Rizzoli Institute by professor Marcacci and Zaffagnini and it is still widely performed in many orthopedic center worldwide.

## Introduction

The anterior cruciate ligament (ACL) is the most commonly injured knee ligament and it is a primary restrain to anteroposterior joint translation as well as rotatory knee laxity. Frequently, other ligamentous structures or the menisci are affected leading to further dynamic instability. Since the first historical attempts in the ‘20 s to surgically restore native knee stability, big steps forwards have been made in terms of anatomical knowledge, diagnosis of combined lesion, techniques of reconstruction, and postoperative protocols. However, even today there is still a subgroup of patients who continue to experience instability even after an uneventful ACL reconstruction. The rediscovering of the anterolateral ligament (ALL) [1] and its anatomical role in restraining the pivot shift movement has shed light that additional surgical procedures on the anterolateral side of the knee could help the surgeon to improve the surgical outcomes. Based on this evidence, surgical extra-articular augmentation has been advocated by many authors to treat or prevent residual instability.

In this paper we will present with an evidence based approach, the biomechanical rationale, biological evidence, and the long term results of a technique developed more than 25 years ago at the Rizzoli Institute by professor Marcacci and Zaffagnini that has been performed in more than 6′000 patients [2].

## Historical background

Since Robert Adams described the first ACL tears in 1837, many steps forward have been made in terms of discovering the ligament anatomy and function, in terms of diagnostic accuracy and many surgical techniques have been proposed for restoring normal knee function [3].

After the first pioneers attempted to reconstruct the ACL in the first decade of the twentieth century, hundreds of different grafts or surgical procedures have been proposed, most of them were surgical fashion abandoned, while others are still performed with obviously some technical changes.

John Marshall is one of the first authors to describe the “Over the top” position during an ACL reconstruction. In 1979 he published the “Quadriceps tendon substitution technique” that consisted in harvesting the patellar ligament and part of the quadriceps tendon as a graft, that was pulled through a tibial tunnel and then secured “over the top” of the lateral femoral condyle [4]. James Horne from Canada had some concerns regarding the femoral tunnel position of the graft and proposed to follow an anatomical line from the tibial tunnel and again, fasten it over the lateral femoral condyle [5].

The idea that an extra-articular procedure could improve the outcome of the ACL surgery and reduce the disability of this lesion should be dated back to Henry Milch who wrote in 1935 that “an ACL-deficient knee will have little instability if no associated injuries to the collateral ligaments are present”. Years later the rationale for an associated lateral procedure was elucidated by Ellison with the sentence “it is easier to control the rotation of a wheel at its rim than at its hub” [6].

**Fig. 1:**
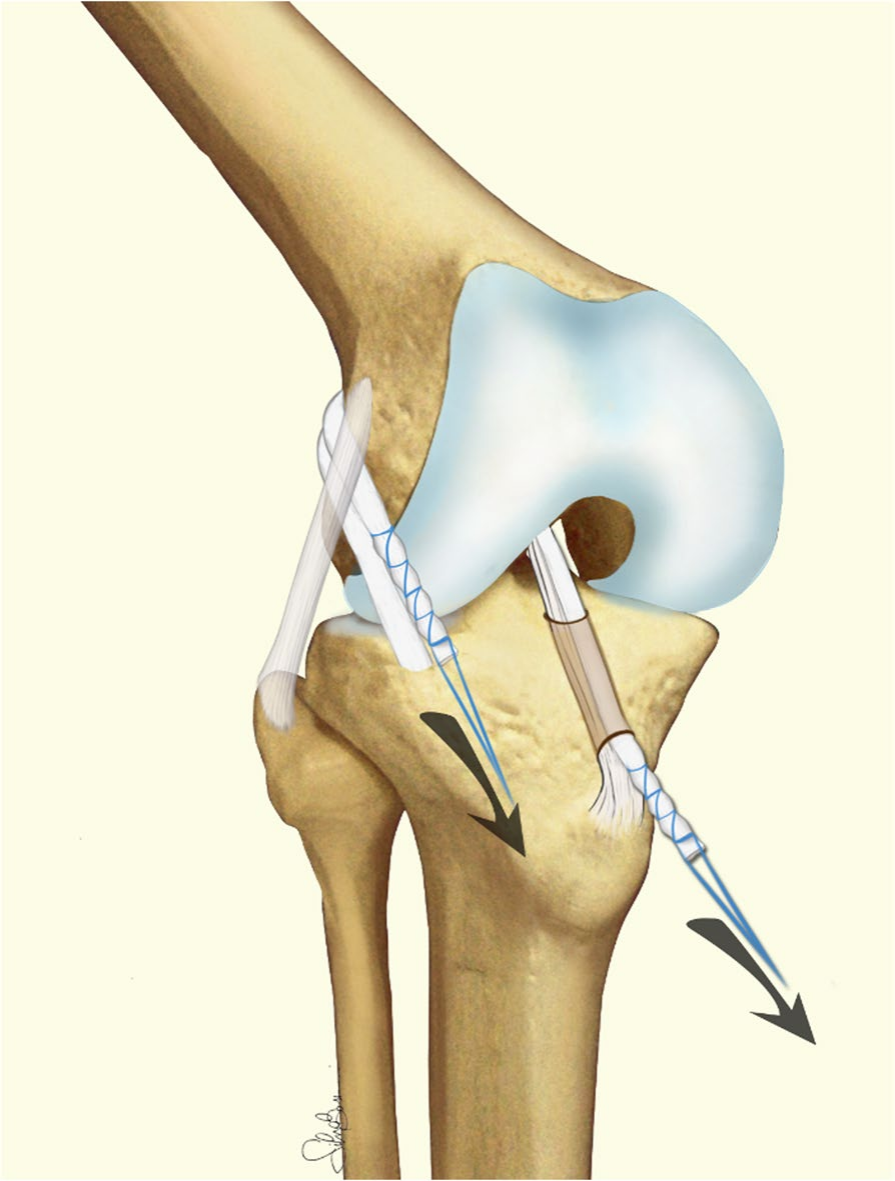
Surgical technique reconstruction for ACL and lateral plasty described by Zarins and Carter Rowe

The first clinical report of a large group of patients treated with a technique similar to the “Single Bundle Over The Top” was published in 1985 by Bertrand Zarins and Carter Rowe, from the Harvard Medical School of Boston (Fig. 1) [7]. Their technique consisted of an associated intra and extra-articular reconstruction: the semitendinosus tendon and the ileo-tibial tract were routed from opposite directions over the top of the lateral femoral condyle and then were passed through the same tibial tunnel. Of the 100 patients evaluated at the follow-up, in 80 and 94 cases, the Lachman and the Pivot shift test was reduced to zero or 1 + . The authors reported also that the isokinetic muscle performance and the passive tibial rotation were also improved from the preoperative condition.

Nowadays, the most common extra-articular procedures performed are Lemaire [8], Ellison [9], Cocker Arnold [10], and MacInthosh [11] reconstruction. These procedures are performed in combination with an intra-articular reconstruction and have been developed and slightly modified during the last decades. Another associated intra- and extra-articular reconstruction that was developed in those years is the Single Bundle Over-The-Top technique [2], which will be presented in this manuscript.

## Anatomical background

### Intra-articular reconstruction

**Fig. 2:**
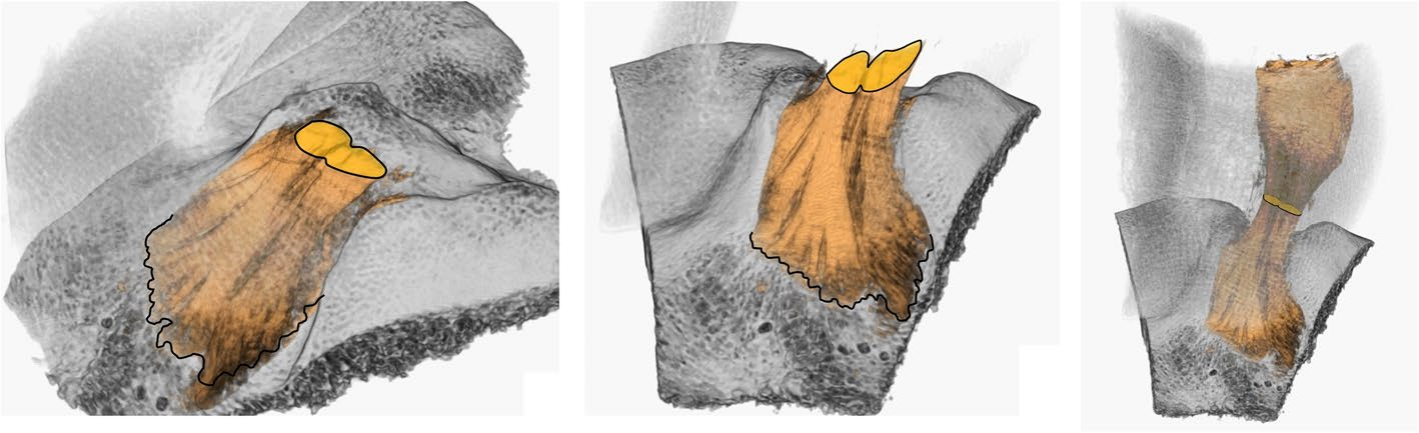
3D-CT of the tibial insertion of a human ACL

The ACL is composed macroscopically of two different bundles, the Anteromedial (AM) and the Posterolateral (PL), so named based on the relative insertion on the tibial surface [12] (Fig. 2).

The native ACL femoral footprint is a very large area with a mean long axis of 17.7 mm and covers an area of 115-230mm^2 ^[13, 14]. The surgical goal of the ACL reconstruction could not be to reproduce completely the anatomy of the femoral footprint, because, even an oversized single 12mm^2^ tunnel will cover only the 66% of the footprint, and a double-bundle with two 6mm^2^ tunnels will reproduce just the 71% of this surface [15]. Moreover, there is a discrepancy between the size of the femoral ACL insertion and the morphology of the ligament itself, the concept of “filling the footprint” with ACL graft does not reproduce the morphology and the kinematical behavior of the native ACL [16].

As it is important to restore the native kinematic behavior, the surgeon must know the histological properties and the biological environment of the ACL as well. In the specific, Iwahashi et al. [17] described two different types of ACL insertion: the “indirect fibers” where the ligament directly anchors to the bone with Sharpey-like fibrils, and the “direct fibers” whose ultrastructure of dense collagen and a transitional fibrocartilage zone suggest a more important load-bearing function and consequently, their role in force distribution at different degrees of tensioning. Pathare et al. [18] performed an arthroscopic debridement of the “indirect fibers” in cadaveric knees observing a minimal increase in anterior tibial translation (ATT = 0.37 mm) and rotational translation after simulated pivot-shift. While the whole ACL resection (indirect and direct fibers) carried more than a tenfold increase in ATT (4.44 mm) and during pivot shift, suggesting a more biomechanically relevant role of the “direct fibers “ [18]. In similar settings, Kawaguchi et al. [19] and Nawabi et al. [20] confirmed and reinforced these findings: the “high fibers” located near the roof of the intercondylar ridge (very close to the “Over the Top” position) carried 82–95% of the load during anterior-drawer tests and 84% of the load during simulated pivot-shift at 15° of flexion.

**Fig. 3:**
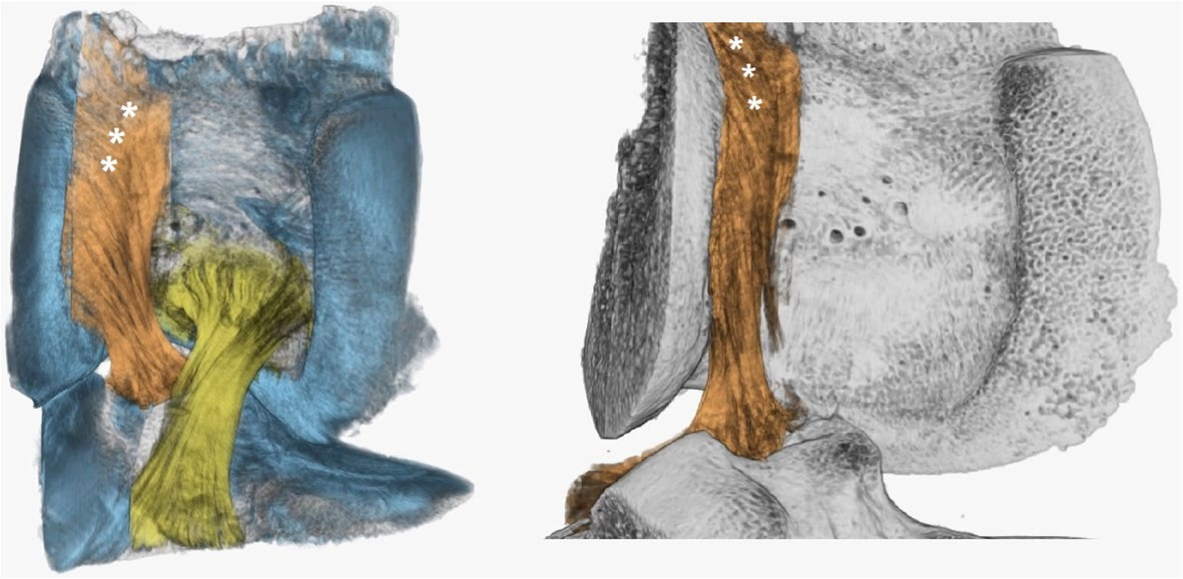
3D- CT of the intercondylar groove. The ACL (orange) is composed of a multitude of fibers. The stars indicate the direct fibers, directed in the “Over the top” position

Focusing on the real aim of ACL surgery, Pearle et al. [16] described the concept of the “I.D.E.A.L.” femoral position in order to achieve satisfactory results. “I.D.E.A.L.” means that firstly the graft should have a length-tension relationship similar to the native ACL [21] which is typically low [22]. In fact, it has been demonstrated that the tunnel incorporation is sensitive to dynamic changes in ACL graft force during the range of movement and that high stresses on the graft impair the graft-tunnel osseointegration. Respecting this principle will avoid graft failure by overstretching [23]. Then the surgeon must take into account the “direct fibers” [14], their anatomical position, and the eccentric graft placement, which means a position anterior or higher in the native femoral footprint [24] (Fig. 3).

Given all these considerations and principles, it is clear that ACL surgery represents a compromise: the surgical goal should be to restore normal biomechanics by an isometric reconstruction. This result could be achieved even with the so-called “non-anatomic” techniques. Sundemo et al. [25] have demonstrated in a cohort of 193 patients at 16 years of follow-up that a non-anatomic reconstruction if performed isometrically, did not predict the long-term subjective outcome, functional outcome, or the development of osteoarthritis.

### Extra-articular lateral plasty

A renewed interest in anterolateral structures and their clinical relevance has grown after the studies published by Claes et al. in 2013 [1]. The authors described a ligament on the anterolateral part of the knee (ALL) “clearly distinguishable from the lateral capsule and definitely separated from the iliotibial band” [1]. These findings helped to ignite a heated debate within the scientific community. After 5 years of discussions, a panel of renowned researchers and surgeons have concluded: not only the ALL exists [26], but also provides significant rotatory stability and acts as a secondary stabilizer to the ACL.

In the past, many other authors, starting from Paul Segond [27] in 1879 have described a similar ligamentous structure on the anterolateral part of the knee. However, its biomechanical role has been described almost one century later, in 1976, when Hughston et al. published the “Classification of knee ligaments instabilities”, a paper that represents a milestone. They described the “anterolateral rotatory instability” as a consequence of the tear of the middle third of the lateral capsular ligament [28].

A few years later, Terry et al. [29] described a biomechanical synergism between the ACL and the so-called “capsulo-osseous layer” of the Iliotibial band. These two structures together form an inverted “U” that surrounds the lateral femoral condyle and contribute to rotatory stability [30]. For the author, the ACL alone was not responsible for the variations of instability clinically observed, while combined injuries with the deep ileo-tibial tract were correlated with the different degrees of the Lachman and Pivot Shift test. In fact, Norwood et al. in a series of 36 patients with acute anterolateral instability, reported only 4 patients with an isolated ACL lesion, while in all the remnant cases, structures on the lateral side of the knee were involved [31].

Nowadays, many studies have been published following these pioneering authors of the last century. In a recent review, 53 recent studies that discuss the ALL were identified, this suggests the renewed attention of the scientific community to the periphery of the knee and the extra-articular procedures as a possible resource to improve the ACL-reconstruction outcome [32].

## Technical considerations – focus on tips and tricks

**Fig. 4:**
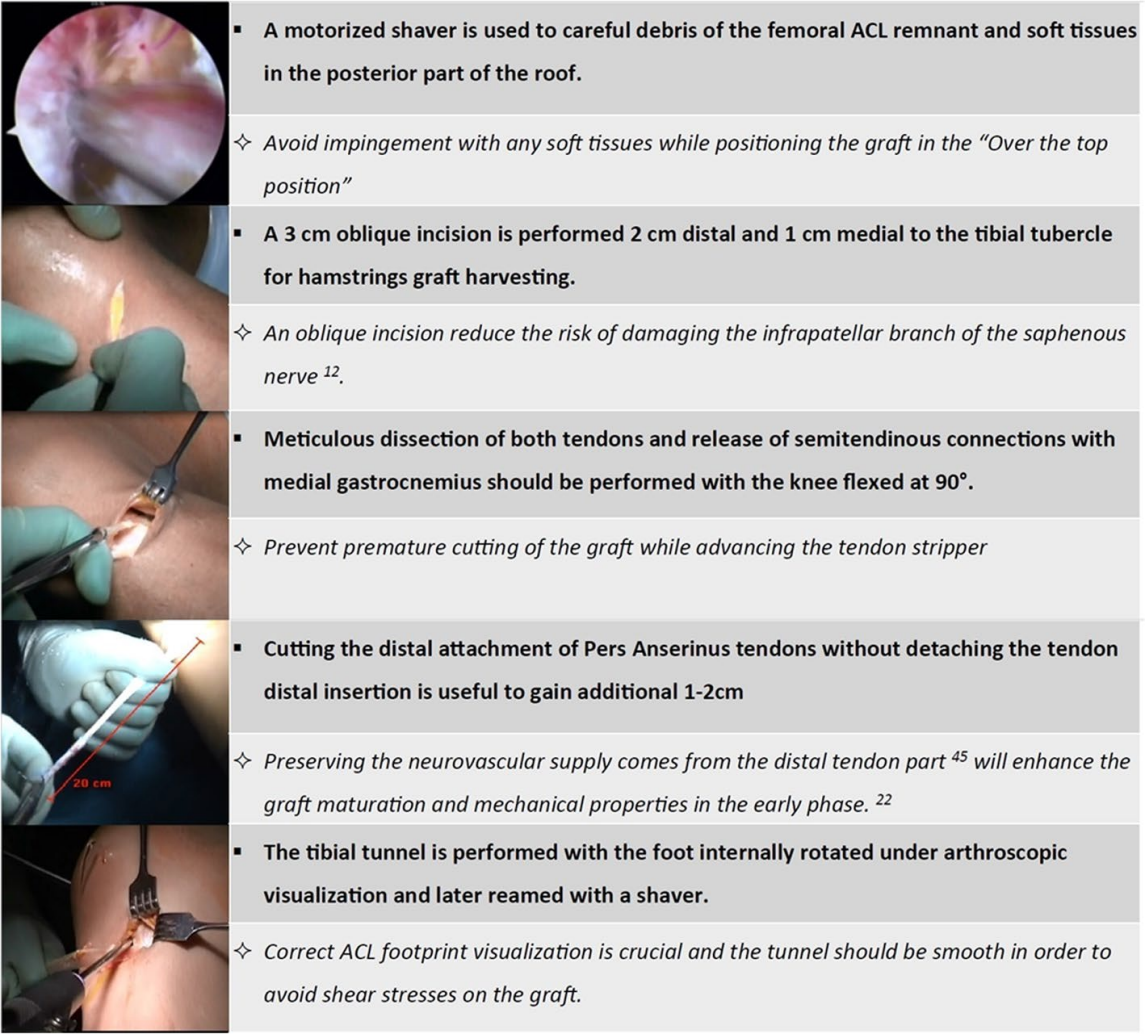
Technical tips for over-the-top and lateral plasty ACL reconstruction

**Fig. 5:**
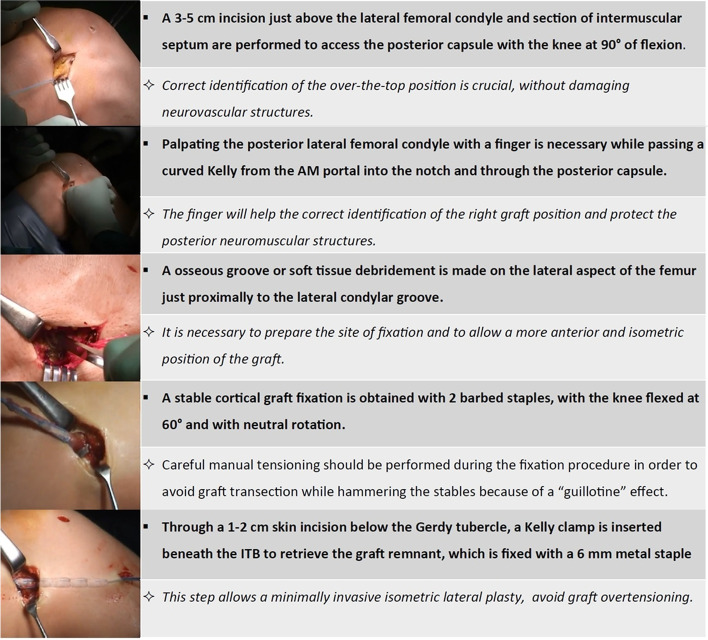
Technical tips for over-the-top and lateral plasty ACL reconstruction

The surgical technique is broadly described in the original paper from 1998 [2]. The tourniquet is inflated, and a diagnostic arthroscopy is performed to treat eventual cartilage or meniscal pathologies and clean the ACL femoral insertion. After tendons harvesting preserving the tibial attachment, the tibial tunnel is drilled (usually 7 mm) (Fig. 4) and a straight lateral incision is performed on the lateral aspect of the knee proximal to the lateral epicondyle. By splitting the intermuscular septum, it is possible to reach the posterior aspect of the joint capsule, where a hole is performed with the tip of a curved Kelly clamp inserted in the anteromedial portal. In this way, using shuttle sutures, it is possible to pass the graft through the tibial tunnel, inside the joint, and then outside the joint from the lateral incision. The graft is tensioned manually and secured in the “over the top” position using two 8 mm metal staples with the knee flexed to 60° and neutral rotation. Finally, the remaining part of the graft is passed deep to the iliotibial band, superficial to the lateral collateral ligament, and is fixed below the Gerdy’s tubercle with a 6 mm metal staple as extra-articular plasty (Fig. 5).

A knee brace is not used postoperatively. Range of motion, quadriceps muscle active exercises, straight-leg raises, and prone hamstring muscle-stretching exercises are all begun the day after surgery. Functional muscle stimulation is used for 2 h, 3 times daily, for 4 weeks. Patients are allowed partial weight-bearing during the first 2 weeks, while full passive extension and active flexion through the range from 0° to 120° is started from the third postoperative day. Three weeks after surgery, full weight-bearing is allowed. Stationary biking, active knee extension with weights, and one-quarter squats are introduced 4 weeks after surgery. Running is started at 3 months and pivoting sports activities after 6 months.

## Biomechanical considerations

In order to demonstrate the kinematic reliability of the “Over the top” ACL reconstruction, several biomechanical studies both cadaveric and in vivo have been performed.

In 2009, Bignozzi et al. [33] quantified in vivo the reduction of anterior–posterior translation obtained by the lateral plasty added to the intra-articular single bundle (SB) reconstruction. Twenty-eight patients with isolated ACL injury underwent navigated ACL reconstruction with over-the-top and lateral plasty, and a kinematic analysis was performed after each step of the surgical procedure. Compared to the pre-operative status, the intra-articular procedure alone was able to reduce the anterior translation of lateral compartment by 5 mm, while the association of the lateral plasty further decreased the laxity by 1.6 mm at 30° and by 1.0 mm at 90° of flexion.

In 2017, Bonanzinga et al. evaluated the effect of over-the-top and lateral plasty ACL reconstruction in the setting of combined ACL and ALL lesion through a cadaveric model. The authors, using a computer navigation system, showed that ALL sectioning significantly increased internal tibial rotation and pivot shift acceleration of the ACL-deficient knee, however without affecting antero-posterior laxity. Then, ACL reconstruction with over-the-top and lateral plasty was able to better reduce the internal rotation and pivot-shift acceleration in respect to a non-anatomic Double Bundle (DB) technique [34], however without creating joint overconstrain. [2]. These results suggest that an intra-articular reconstruction alone may not be sufficient to address combined rotatory instability when ACL rupture is combined with an antero-lateral injury. Considering that recent studies have identified an incidence of combined ALL and ACL lesion in 30–40% of the cases, this subgroup of patients may benefit from an associated extra-articular procedure even in the context of primary reconstruction [35].

Finally, in 2019 Grassi et al. [36] compared the biomechanical results of three different ACL Reconstruction techniques: 42 patients were randomized to receive ACL surgery either with a hamstring quadrupled isolate Single Bundle, a Double Bundle or a Single Bundle plus lateral plasty technique. The results showed that, even in vivo, the over-the-top and lateral plasty was the most effective in controlling internal–external rotation and the anterior–posterior translation during the Lachman test and reduced AP laxity at 90° of flexion compared with the pre-operative state, moreover its results were superior when compared with quadrupled isolate SB and DB.

## Biological considerations

**Fig. 6:**
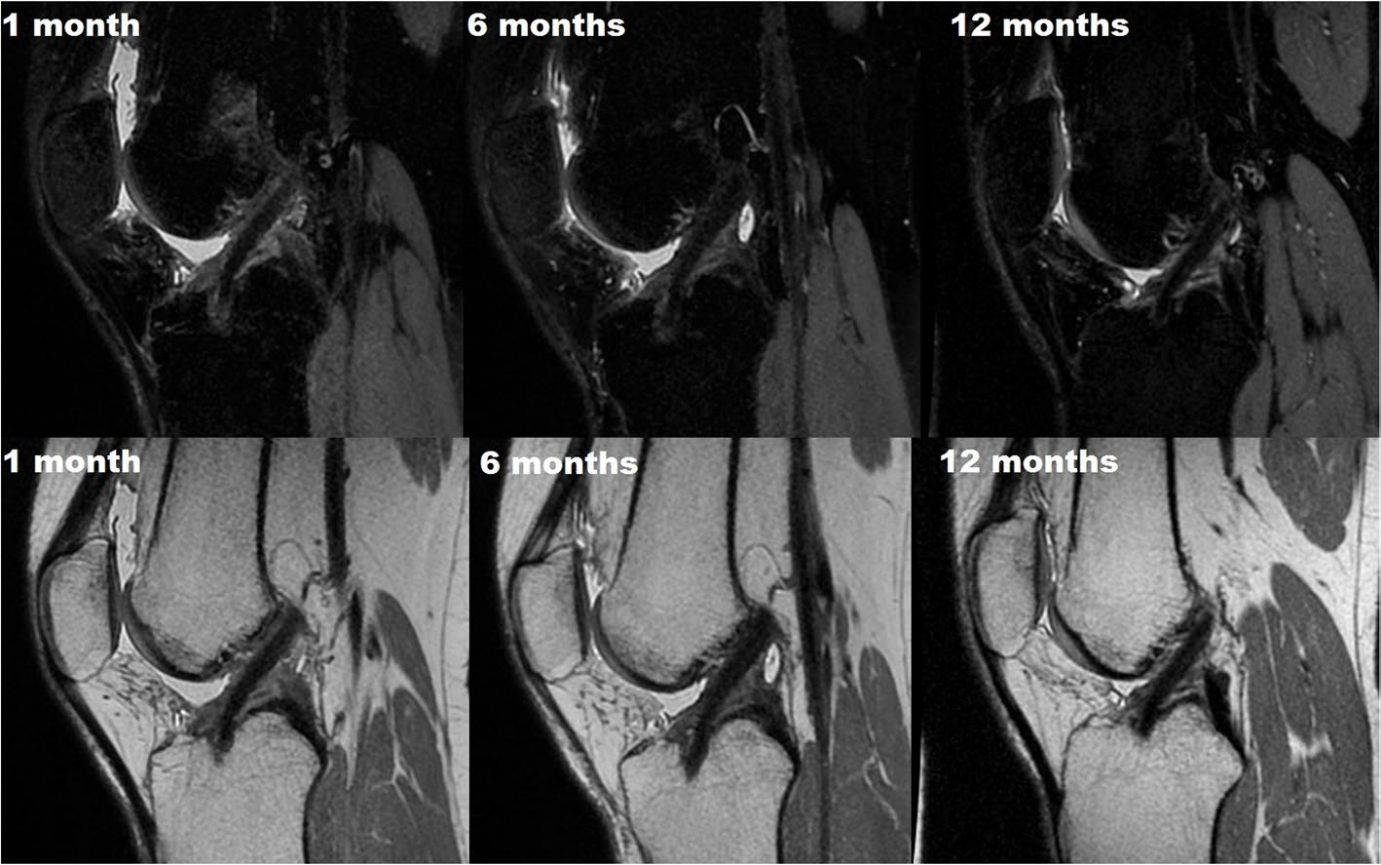
MRI of hamstring ACL graft that was harvested preserving the distal portion 1, 6, and 12 months after surgery

One of the main features of the present over-the-top ACL reconstruction with lateral plasty is the possibility to leave the tibial insertion of the hamstrings intact. From a biological point of view, this represents a great advantage, which is supported also by cadaveric studies, animal models, and clinical series (Fig. 6).

In 2002 Zaffagnini, Golano and other researchers performed an elegant dissection and histological study aimed to evaluate the vascularity and neuroreceptors of the Pes Anserinus [37]. The authors demonstrated that the Pes Anserinus insertion is well vascularized and richly innervated and that these morphological features continue along the length of the tendons. In fact, a widespread array of small vessels from the arterial arch that receives its blood supply from three main arteries of the knee (inferior medial genicular artery, inferior lateral genicular artery, anterior tibial recurrent artery) were observed to enter the gracilis and semitendinosus tendons ascending from the periosteum at their insertions along the tendons. The number and vessel diameter decrease from 2,201 μm at the osteotendinous part to 661 μm in the middle part of the tendon about 8 cm from the insertion site, but with no avascular regions. Histologic samples demonstrated also the presence of a wide array of nerve fibers, Ruffini corpuscle, and other non-encapsulated sensory nerve endings. The well-developed vascularization of the tibial insertion of the Pes Anseriuns tendons stands in contrast to the avascular nature of the Patellar Tendon, which receives blood supply from the peripatellar plexus and with no contribution through its tibial insertion [37].

The biological advantages of the neuro-vascular supply of the Pes Anserinus insertion have been investigated also in an animal study on 64 New Zeland rabbits, demonstrating the ability to bypass the graft avascular necrosis stage, resulting in better tendon-bone healing and improved mechanical properties [38]. Specifically, in the group of rabbits where the ACL was reconstructed preserving the distal insertion of the semitendinosus, no evident necrosis or hypocellularity was observed in the graft within the tunnel from 3 to 24 weeks after surgery. Moreover, the early formation of Sharpey-like fibers, the early proliferation of mineralized fibrocartilage in the tendon-bone interface, and the early presence of an organized insertion-like architecture at 24 weeks were noted. Differently, when the graft was detached, the three characteristic stages of necrosis, proliferation, and maturation were noted at histologic evaluation. Furthermore, tunnels were characterized by wider area, the presence of fibrovascular “scarring” tissue at the tendon-bone interface, and lower-density bone deposits around the tunnel wall at micro-CT evaluation. The “biologic superiority” of the tibial insertion preservation approach was practically expressed in a significant improvement of biomechanical properties of the graft; in fact, higher load to failure and stiffness were measured at 12 and 24 weeks.

The interesting findings of the animal model were confirmed also in a clinical setting since Liu et al. [38] reported an enhanced graft maturation when ACL reconstruction was performed preserving the Pes Anserinus insertion compared to hamstrings detachment. The authors showed a stable low-intensity signal after reconstruction throughout serial MRI evaluation up to 24 months when hamstring insertion was maintained. Differently, patients treated with the free graft exhibited a course of initial increase of graft signal with a peak at 6 months, followed by a gradual decrease until 2 years. Moreover, a significantly higher signal was measured in the “critical” return-to-sport period between 6 and 12 months.

## Twenty-five years of clinical results

Clinical outcomes of the over-the-top and lateral plasty have been extensively studied in the last 25 years. In the first report, dated 1998, Marcacci et al. [2] evaluated the first group of 40 patients with a minimum 2-year follow-up operated since 1992. The authors described a normal or nearly normal knee in 92.5% of patients, a mean Lysholm score of 95 points, a flexion deficit of 6°-15° in only 2 patients, and no cases of flexion contracture. The antero-posterior knee stability was satisfactory, with a mean side-to-side difference measure with KT-2000 of 2.1 mm and 93.3% of patients with values between 0 and 5 mm. Finally, a return to pre-injury level was possible in 90% of cases. In 2003, the same authors reviewed the 5-year follow-up of 60 patients, reporting similar results [39]. Meanwhile, the over-the-top reconstruction has been also studied in several randomized studies to assess its efficacy in comparison with other techniques. The 3-year outcomes were similar to those of a non-anatomic double-bundle technique in terms of knee stability and patient satisfaction [2].

At 5 years, 75 patients randomized in three different techniques exhibited higher subjective scores and faster return to sport when treated with over-the-top and lateral plasty technique in respect to four-strand hamstrings and patellar tendon grafts. Moreover, a lower rate of positive pivot-shift was found compared to four-strand hamstrings, and fewer cases of anterior knee pain or ROM limitations were reported compared to the patellar tendon. Finally, no relevant tunnel enlargement was noted at radiographic evaluation [40].

**Fig. 7:**
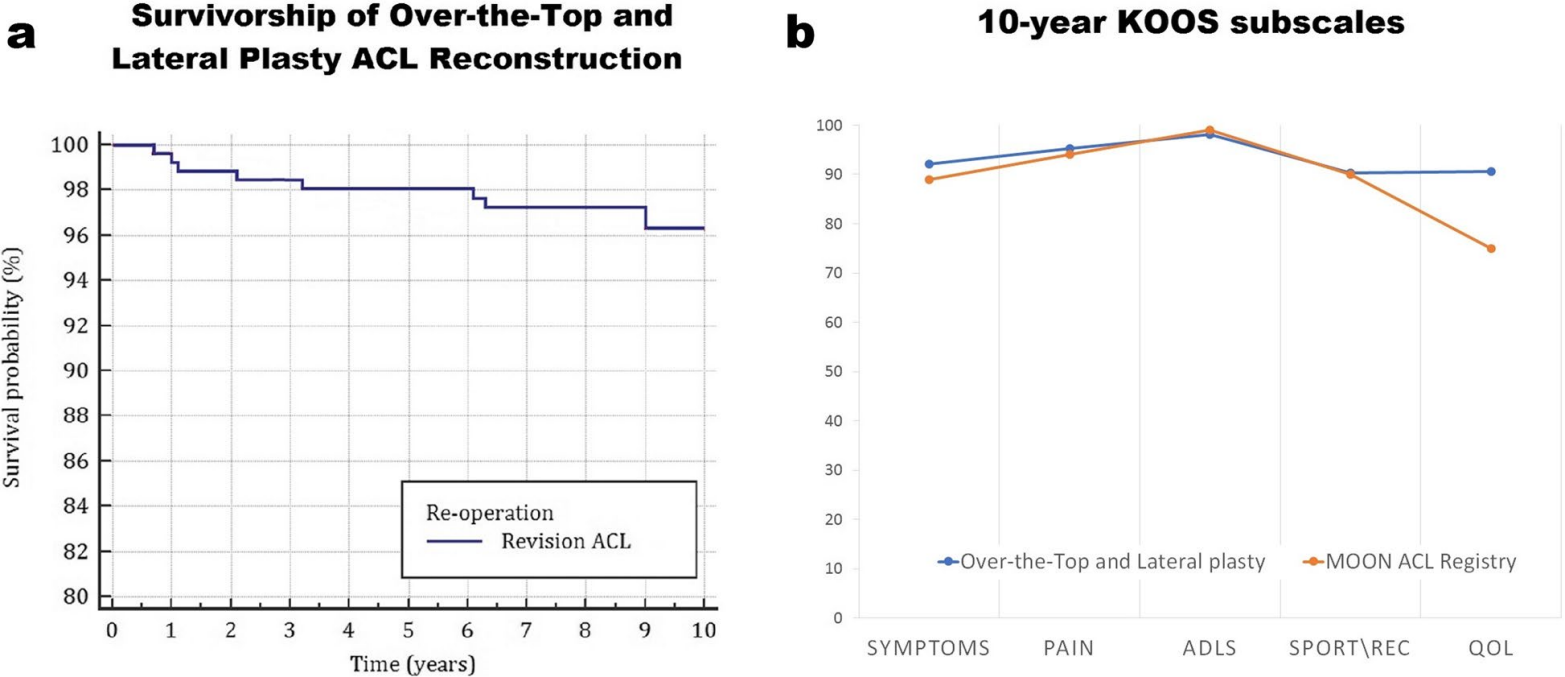
**a-b** Survival curve of ACL over the top and lateral plasty ACL reconstruction at 10 years (**a**); 10 year KOOS Subscale of Over-the-top (blue) and MOON ACL Registry (orange) (**b**)

The continuous and systematic use of the over-the-top technique over the years allowed the collection of relevant data regarding its long-term outcomes. The 11-year results of 54 high level sports patients showed subjective and objective results comparable to those of previous short- and medium-term follow-ups, with 90% of patients exhibiting a normal or nearly normal knee, 92% and 89% of patients presenting full extension and flexion respectively, and with a stable knee in 94% of cases. Furthermore, the comparison between 5 and 11 years radiographs showed no progression of tibial tunnel enlargement [41]. Furthermore, the review of 267 consecutive patients with a minimum follow-up of 10 years allowed to determine a revision rate of 1.1% at 2 years, 1.9% at 5 years, and 3.7% at 10 years (Fig. 7a). A total of 13% of patients underwent reoperation, mainly staples removal (5%), while the Patient Reported Outcome Measures (PROMs) evaluated with the KOOS Score were substantially comparable with the 10-year results of the Multicenter Orthopaedic Outcome Network (MOON) ACL registry [42] (Fig. 7b).

A relevant report on the very long-term clinical and radiographic results was published in 2017 [43] where 29 patients of the original case series of patients operated in the’90 were evaluated at a mean follow-up of 24 years. The patients, which had a mean age at surgery of 25.5 ± 7.6 years, presented a normal or nearly normal knee in 86% of the cases. The Lysholm score, which had a mean value of 85.7 ± 14.6, was ranked a good or excellent in 62% of patients. As expected, a gradual decrease was noted with respect to earlier follow-ups. Objectively, 14% had minor ROM restriction and 14% had positive Lachman or Pivot-Shift. Despite the aging, 86% of patients were still involved in sports activities at the final follow-up, mostly low-moderate impact sports at a recreational level. An interesting analysis was performed on knee radiographs; in fact, no signs of lateral osteoarthritis due to the extra-articular plasty were noted, since the joint-space-narrowing was similar between the operated and healthy knee. Regarding the medial compartment, a higher narrowing compared to healthy knee was found only in patients with concurrent medial meniscectomy, thus highlighting the role of the meniscal defect in the progression of osteoarthritis rather than ACL reconstruction.

Finally, to assess the safety profile of the adding and extra-articular procedure to the intra-articular over-the-top reconstruction, the medical charts of 2559 consecutive ACL reconstructions performed in a 7-year period were reviewed and the 90-days re-admission rate was determined. Overall, 2.3% of patients were re-admitted within the first 3 months since ACL reconstruction due to knee swelling (0.78%), superficial infection (0.63%), deep infection (0.55%) or joint stiffness (0.23%) [44]. Therefore, these data confirmed the over-the-top plus lateral extra-articular plasty as a safe technique with a low rate of peri-operative complications.

## Combined procedures and special populations

The over-the-top plus lateral plasty ACL reconstruction has been used with good results also in combination with other surgical procedures and to treat special patient populations.

### Revision ACL reconstruction

The present technique was successfully employed also to treat multiple cases of ACL reconstruction failures where bone-patellar tendon-bone, allografts or synthetic ligaments were used as primary grafts. The avoidance of femoral tunnel drilling typical of the over-the-top technique allowed for bypassing femoral tunnel enlargement or problematic hardware removals [45].

From 2016 to 2018, around 10% of revision ACL performed in our department has been performed with over-the-top and lateral plasty with hamstrings, obtaining satisfactory subjective and objective clinical results. In the case of previous hamstring harvesting, which occurs more frequently, a long Achilles Tendon allograft could be used to replicate the same technique, fixing the tibial side with a metal staple. Also, other authors used a similar the over-the-top technique for revision ACL with successful results [46, 47]. Preliminary data, based on a series of 34 patients who underwent ACL revision with Over-the-top plus lateral extra-articular tenodesis with Achilles tendon allograft at a mean 6.1 years of follow-up, showed a surgical failure rate of 3% (1 patient) and a rate of low rate of reoperations 12% (mostly hardware removal).

### Skeletally immature and adolescent patients

Twenty skeletally immature patients with a mean age of 12.3 years were treated with a modified physeal sparing technique [48]. After a mean follow-up of 54 months, all the patients had KT-1000 side-to-side difference < 3 mm, 19 patients were scored as IKDC A and only one as IKDC B; moreover, 60% returned to the same pre-injury level and 30% were able to perform at a competitive level. Only 3 patients had minor leg length discrepancies, thus confirming the safety and efficacy of the technique also in patients with open physis. The Over-the-Top technique showed really satisfactory clinical results also in high-risk adolescents. A recent report evaluated the outcome of 151 patients younger than 16 years (mean age 14.8 years) showing a revision rate of only 6% and good or excellent Lysholm score was reported in 88% of the cases. Moreover, 91% of them returned to sport and 80% of them were still active at the final follow-up of 6 years [49]. Similarly, another report of 54 adolescents at a minimum follow-up of 10 years showed that those positive clinical results are stable over time with only 3.5% of revision rate and an average Lysholm of 95 points [50]. Finally, Over-the-top with lateral plasty has been reported to be successful also in adolescents with increased tibial slope, which represents a renowned risk factor for ACL reconstruction failure [51]. In fact, the revision rate in patients with a slope higher than 12° remained significantly lower when compared with the 19% of risk of contralateral ACL injuries [51].

### Professional soccer players

In a recent case-series of professional soccer players who underwent ACL reconstruction with hamstring tendon, there were 17 patients who underwent Over-The-Top Single-Bundle with or without lateral plasty. All those patients returned to play after an average of 6.5 months and the first official game was after an average of 8.0 months in the majority of them. At the final follow-up of 12 years, the average Lysholm score was 94.1 points and there was just one failure (1/9) in the isolate single bundle over-the-top, while no failures (0/8) were found in the over-the-top plus lateral plasty group. Interestingly, the contralateral ACL tear rate was significantly higher (21%) compared to the ipsilateral revision rate, suggesting a very satisfying clinical outcome [52].

### Over-50 years old patients

Fifteen adults (10 males, 5 females) with a mean age of 55.2 ± 4.6 years underwent ACL reconstruction with autologous hamstrings and over the-top plus lateral plasty technique. Despite only 20% had no concurrent meniscal lesions (53% and 33% underwent medial and lateral meniscectomy, respectively) and 4/15 had high grade condropathy, only one patient underwent partial knee replacement while the remaining patients rated their knee as excellent in 64% of cases, good in 29% and fair in 7% at 10 years follow-up. Eight patients were still involved in physical activities such as skiing (1), cycling (1), trekking (1), motocross (1), basket or jogging\walking (3), [42].

### Varus malalignment and unicompartmental osteoarthritis

Nineteen patients were treated between 2002 and 2007 combining ACL reconstruction with over-the-top and lateral plasty with closing wedge lateral valgus high tibial osteotomy [53]. The indication was varus malalignment with unicompartmental osteoarthritis and ACL insufficiency. The ACL reconstruction was performed by harvesting the hamstrings before the closing wedge lateral osteotomy; after stabilizing the osteotomy with a Krackow staple, the graft was passed in the tibial tunnel, fixed at the over-the-top position and then below the Gerdy tubercle to complete the lateral plasty. At a mean follow-up of 6.5 years, the VAS for pain improved from 7.0 to 3.8 points, while the subjective IKDC improved from 60 to 74 points.

### Meniscal allograft transplantation

The celerity and reproducibility of the present technique of ACL reconstruction make it suitable for association with demanding procedures such as meniscal allograft transplantation (MAT). Our experience allowed us to evaluate 20 patients that underwent concomitant medial (16 cases) or lateral (4 cases) MAT and ACL reconstruction at a mean age of 43.9 ± 10.3 years [54]. At a mean follow-up of 5.7 years, a significant improvement in pain, function, and sport activity was reported. The mean Lysholm at the final follow-up was 80.2 points and 40% of patients returned to the same pre-injury sport level. Only two patients (10%) underwent reoperation due to traumatic lateral meniscus lesion and for staples removal.

### Unicompartmental knee replacement

Despite this represents an unusual and rare indication, over-the-top ACL reconstruction could be combined with Unicompartmental Knee Arthroplasty (UKA) in the case of advanced unicompartmental osteoarthritis and ACL deficiency. The lack of femoral tunnel avoids the weakening femoral bone, while the narrow and vertical tibial tunnel does not excessively damage the tibial plateau under the prosthetic component. This combined technique, applied in a few cases, produced good knee stability and patient satisfaction; moreover, no intraoperative or post-operative complications were reported [55].

## Conclusions

Among several surgeries developed for ACL reconstruction, the “Over the Top” technique is a reliable surgery that has been continuously investigated with kinematic testing and clinical high-level studies, even at very long-term follow-up. Biomechanically it ensures good results even if compared with more anatomical and double-bundle reconstructions. Some of the advantages of this technique include the isometric placement of the graft and the absence of a femoral tunnel, and thus, the risk of tunnel convergence during combined intra and extra-articular reconstruction [56] or tunnel malposition, which is considered the principal responsible factor for surgical failure [57–59].

For the same reasons, it represents a good option in cases of ACL revision, multiligament injuries, and in the skeletally immature patients. Finally, it is a reliable and cheap surgery, because it does not require a dedicated instruments or expensive hardware.

## Abbreviations

ACL: Anterior cruciate ligament
ALL: Antero lateral ligament
AM: Anteromedial
PL: Posterolateral
ATT: Anterior tibial translation
SB: Single bundle
DB: Double bundle
PROMs: Patient reported outcome measures
MOON: Multicenter Orthopaedic Outcome Network
IKDC: International knee documentation committee
VAS: Visual analogue scale
MAT: Meniscus allograft transplant
UKA: Unicompartmental knee arthroplasty
